# Erratum: Tiwari, S., et al. Biosensors for Epilepsy Management: State-of-Art and Future Aspects. *Sensors* 2019, *19*, 1525

**DOI:** 10.3390/s19153288

**Published:** 2019-07-26

**Authors:** Shivani Tiwari, Varsha Sharma, Mubarak Mujawar, Yogendra Kumar Mishra, Ajeet Kaushik, Anujit Ghosal

**Affiliations:** 1 Department of Chemistry, School of Basic and Applied Sciences, Galgotias University, Greater Noida, Gautam Buddh Nagar, Uttar Pradesh 201308, India; 2 School of Life Sciences, Jawaharlal Nehru University, New Delhi 110067, India; 3 Bio-MEMS and Microsystems Laboratory, Department of Electrical and Computer, Engineering, Florida International University, Miami, FL 33174, USA; 4 Functional Nanomaterials, Institute for Materials Science, Kiel University, Kaiserstr 2, D-24143 Kiel, Germany; 5 Department of Immunology and Nano-Medicine, Institute of NeuroImmune Pharmacology, Center for Personalized Nanomedicine, Herbert Wertheim College of Medicine, Florida International University, Miami, FL 33199, USA; 6 School of Life Sciences, Beijing Institute of Technology, Beijing 100081, China; 7 College of Chemistry and Materials Science, Northwest University, Xi’an 710069, Shaanxi, China

The authors wish to make the following correction to the above-mentioned published paper [1]:

On page 26, there is correction needed in reference [115]:

“Saleem, W. Biomarkers for Brain Disorders Electrochemically Detected by BRODERICK PROBE^®^ Microelectrodes/Biosensors. *J. Biosens. Bioelectron. S* **2013**, *12*, 2.

This should be replaced with:

“Saleem, W.; Broderick, P.A. Biomarkers for Brain Disorders Electrochemically Detected by BRODERICK PROBE^®^ Microelectrodes/Biosensors. *J. Biosens. Bioelectron. S* **2013**, *12*, 2.”

The authors wish to apologize for any inconvenience caused to the readers by this change.
